# Cocatalyst designing: a binary noble-metal-free cocatalyst system consisting of ZnIn_2_S_4_ and In(OH)_3_ for efficient visible-light photocatalytic water splitting†

**DOI:** 10.1039/c7ra12586k

**Published:** 2018-01-29

**Authors:** Jinyan Zhao, Xiaoming Yan, Ning Zhao, Xiao Li, Bin Lu, Xuhong Zhang, Haitao Yu

**Affiliations:** College of Chemistry and Materials Science, Hebei Normal University Shijiazhuang 050024 China xuhongshd@126.com haitaoyu@hebtu.edu.cn; Institute of Coal Chemistry, Chinese Academy of Sciences Taiyuan 030001 China

## Abstract

A binary noble-metal-free cocatalyst consisting of ZnIn_2_S_4_ and In(OH)_3_ was developed *via* a facile one-step hydrothermal method. The ZnIn_2_S_4_/In(OH)_3_ modified ZnWO_4_ nanocomposite exhibited enhanced photocatalytic H_2_ evolution activity compared to all the related pure samples and binary composite photocatalysts under visible light irradiation. The enhanced photocatalytic hydrogen production activities can be attributed to the synergistic effects of the favorable light trapping ability and efficient spatial charge separation. The photocatalytic hydrogen evolution activity over other semiconductors, such as Zn_2_SnO_4_ and TiO_2_, can also be significantly increased by loading ZnIn_2_S_4_/In(OH)_3_ as a cocatalyst. The results clearly demonstrated that ZnIn_2_S_4_/In(OH)_3_ is discovered as a new class of earth-abundant cocatalyst for water-splitting under visible light irradiation. It is expected that our work could provide a new strategy to improve the visible light response of semiconductors and facilitate their application in water splitting.

## Abstract 2

The ZnIn_2_S_4_/In(OH)_3_ modified ZnWO_4_ nanocomposite exhibited enhanced photocatalytic H_2_-evolution activity.

## Graphical abstract

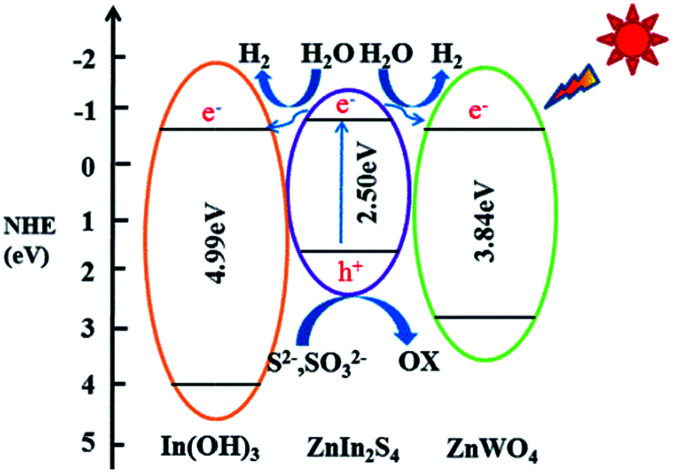

## Introduction

Photocatalytic water-splitting is a promising approach for the clean, economical, and environmentally benign production of hydrogen by utilizing solar energy.^1–8^ During the past few decades, many photocatalytic materials, such as sulfide-, oxide- and oxynitride based materials, have been extensively explored for hydrogen generation through splitting water.^9–11^ However, none of them can meet the requirements for practical large-scale application to photocatalytic H_2_ evolution under visible light. Hence, the highly efficient visible light photocatalysts for hydrogen production is still highly demanded for practical applications.

It is well-known that a cocatalyst plays a significant role in improving both the activity and stability of semiconductor photocatalysts. Noble metals such as Pt, Rh, Au and oxides such as RuO_2_ are generally used as cocatalysts for photocatalytic hydrogen evolution. However, the above noble-metal based cocatalysts are too scarce and expensive. It is necessary to explore alternative cocatalysts based on inexpensive transition metals to facilitate the up-scaling of renewable H_2_ production.

Recently, ZnIn_2_S_4_ with a suitable band gap (2.06–2.58 eV),^12,13^ have been demonstrated to be excellent cocatalyst for CdIn_2_S_4_ and K_2_La_2_Ti_3_O_10_ in the photocatalytic hydrogen evolution.^14–16^ It has been also proved to be an efficient visible-light driven photocatalyst or sensitizer to extend the absorption spectra of some wide-gap photocatalysts from the UV region to the visible region.^17,18^

Furthermore, the unique 2D layer structure nanostructure of ZnIn_2_S_4_ enables its application as an excellent supporting matrix in the construction of heterostructured nanocomposite photocatalysts with high photocatalytic activity.^19–22^ In addition, it was reported that indium ions can hydrolyze into In(OH)_3_ when the pH value of the reaction system was above 2.5.^23^ So it is convenient to obtain ZnIn_2_S_4_/In(OH)_3_ nanocomposite by a simple hydrothermal strategy by adjusting the pH value of the solution. In(OH)_3_ is an important wide-gap semiconductor material with a direct bandgap of 5.15 eV.^23–25^ The wide band gap provides photogenerated charge with stronger redox capability, favoring photocatalytic performance. Therefore it is expected that ZnIn_2_S_4_/In(OH)_3_ can be an ideal candidate as a non-noble-metal cocatalyst system for water splitting under visible light irradiation.

Herein, the ZnIn_2_S_4_/In(OH)_3_ modified ZnWO_4_ nanocomposite was fabricated by a facile three-step hydrothermal strategy. The results revealed that as-prepared ternary ZnIn_2_S_4_/In(OH)_3_/ZnWO_4_ nanocomposite exhibited enhanced photocatalytic H_2_-evolution activity than all the related pure samples and binary composite photocatalysts. Based on the experimental results, a possible synergistic mechanism for the enhanced photocatalytic H_2_ evolution activity was proposed. The photocatalytic hydrogen evolution activity over other semiconductor such as Zn_2_SnO_4_ ^26^ and TiO_2_,^27^ can also be significantly increased by loading ZnIn_2_S_4_/In(OH)_3_ as a cocatalyst. The results clearly demonstrated that ZnIn_2_S_4_/In(OH)_3_ is discovered as a new class of earth-abundant cocatalyst for water-splitting under visible light irradiation. It is expected that our work could provide deep insight into design and application of new system with high activity for solar hydrogen generation in the future.

## Experimental

### Preparation of photocatalysts

#### Synthesis of ZnIn_2_S_4_, In(OH)_3_ and ZnIn_2_S_4_/In(OH)_3_

ZnIn_2_S_4_ was synthesized by a hydrothermal method. In a typical procedure, 0.300 g Zn(NO_3_)_2_·6H_2_O, 0.782 g In(NO_3_)_3_·4.5H_2_O and 0.616 g thiourea were dissolved in deionized water. The pH value of the solution was adjusted to 1.00 by adding 0.1 mol L^−1^ HCl. The mixed solution was then transferred into a 100 mL Teflon-lined autoclave and heated at 180 °C for 12 h. After being cooled to room temperature, the yellow precipitate was collected by centrifugation and washed with ethanol and the distilled water for three times, respectively. And then it was dried at 80 °C to obtain the product.

The preparation of In(OH)_3_ was similar to that of ZnIn_2_S_4_. In a typical procedure, 0.782 g In(NO_3_)_3_·4.5H_2_O was added into the solution. The pH value of the solution was adjusted to 6 by adding 0.2 mol L^−1^ NaOH. The mixed solution was then transferred into a 100 mL Teflon-lined autoclave and heated at 180 °C for 12 h. The remaining steps are the same as ZnIn_2_S_4_.

The synthesis of ZnIn_2_S_4_/In(OH)_3_ is similar to that of ZnIn_2_S_4_. All of the experimental conditions are same except the initial pH value. The pH value of the solution was adjusted to 3.5, 4.5 and 5.5 with 0.2 mol L^−1^ NaOH, respectively. The products synthesized at different pH values were labeled as ZnIn_2_S_4_/In(OH)_3_-3.5, ZnIn_2_S_4_/In(OH)_3_-4.5, ZnIn_2_S_4_/In(OH)_3_-5.5, respectively.

#### Preparation of ZnWO_4_

5 mmol Na_2_WO_4_·2H_2_O and 5 mmol Zn(NO_3_)_2_·6H_2_O were added to 70 mL deionized water with magnetic stirring to form a homogeneous solution. The reaction mixture was then sealed in a 100 mL Teflon-lined autoclave and kept at 180 °C for 12 h. After cooling, the product was filtered, washed, and dried at 80 °C.

#### Fabrication of the ZnIn_2_S_4_/ZnWO_4_ nanocomposite

0.2 g ZnIn_2_S_4_ was ultrasonicated in 70 mL deionized water for 20 min. After that, 0.15 g ZnWO_4_ was added into the solution to prepare the ZnIn_2_S_4_/ZnWO_4_ composite. The mixture was continued to be ultrasonicated for 20 min to obtain a homogeneous suspension and then it was stirred vigorously. Next, this suspension was transferred into a 100 mL Teflon-lined autoclave and maintained at 180 °C for 12 h. After cooling, the product was filtered, washed, and dried at 80 °C.

#### Preparation of In(OH)_3_/ZnWO_4_ nanocomposite

0.2 g In(OH)_3_ was ultrasonicated in 70 mL deionized water for 20 min. After that, 0.15 g ZnWO_4_ was added into the solution to prepare the In(OH)_3_/ZnWO_4_ composite. The mixture was continued to be ultrasonicated for 20 min to obtain a homogeneous suspension and then it was stirred vigorously. Next, this suspension was transferred into a 100 mL Teflon-lined autoclave and maintained at 180 °C for 12 h. After cooling, the product was filtered, washed, and dried at 80 °C.

#### Synthesis of ZnIn_2_S_4_/In(OH)_3_/ZnWO_4_ (ZIZ) nanocomposite

0.2 g ZnIn_2_S_4_/In(OH)_3_ synthesized at different pH values was ultrasonicated in 70 mL of deionized water for 20 min. After that, 0.15 g ZnWO_4_ was added into the solution to prepare a series of ZIZ composites. The mixture was continued to be ultrasonicated for 20 min to obtain a homogeneous suspension and then it was stirred vigorously. Next, this suspension was transferred into a 100 mL Teflon-sealed autoclave and maintained at 180 °C for 12 h. After being cooled to room temperature, the precipitate was collected by centrifugation and washed with ethanol and the distilled water for three times, respectively. And then it was dried at 80 °C to obtain the product. The obtained samples were ZnIn_2_S_4_/In(OH)_3_/ZnWO_4_-3.5, ZnIn_2_S_4_/In(OH)_3_/ZnWO_4_-4.5 and ZnIn_2_S_4_/In(OH)_3_/ZnWO_4_-5.5, which were labeled as ZIZ-3.5, ZIZ-4.5, ZIZ-5.5, respectively.

### Characterization

The phase structure and crystallinity of the products was measured by X-ray diffraction (XRD) patterns, which were recorded on a Bruker D8 Advance X-ray diffractometer with Cu Kα radiation at 40 kV and 40 mA. The morphology of the samples was detected by field emission scanning electron microscopy (FESEM, Hitachi S-4800) and transmission electronic micrograph (TEM, Hitachi, H-7650). High resolution transmission electron microscopy (HR-TEM) images were collected on a JEM-2100 electron microscope, operated at an acceleration voltage of 200 kV. XPS studies were carried out by means of a VG-ESCALAB250 electronic spectrometer using an Al Kα excitation source (*hν* = 1486.6 eV). The position of the C 1s peak was taken as a standard (with a banding energy of 284.6 eV). The Brunauer–Emmett–Teller (BET) surface areas (*S*_BET_) of the samples were measured by a N_2_ sorption analyzer (Quantachrome, NOVA 4000e) at a liquid nitrogen temperature. UV-vis diffuse reflectance spectra of the samples were evaluated using a UV-vis spectrophotometer (U-3010, Hitachi, Japan). Photoluminescence (PL) spectra of the samples were performed using an Hitachi F-4600 fluorescence spectrophotometer.

### Photocatalytic H_2_-production activity

The photocatalytic hydrogen-production experiment was conducted in a closed gas circulation and evacuation system fitted with a top window Pyrex cell. A 300 W xenon lamp (PLS-SXE300C, Beijing Perfectlight Co. Ltd., China) which was positioned 10 cm away from the solution surface, coupled with a UV cut-off filter (*λ* > 420 nm) was used to provide the visible light. The focus intensity measured by a spectroradiometer (PM100D, Thorlabs, America) was 0.54 W cm^−2^. In each run, 0.05 g photocatalyst was dispersed in a 100 mL aqueous solution containing 0.25 M Na_2_SO_3_ and 0.35 M Na_2_S, which served as a sacrificial agent. Before the irradiation with visible light, the suspension was degassed with N_2_ for 1 h to drive away the O_2_ in the system. The reaction cell was kept at room temperature with cooling water. The produced H_2_ was detected using an online gas chromatography (GC7900, N_2_ carrier, 5 Å molecular sieve column, TCD detector). The reaction was continued for 3 h. The apparent quantum efficiency (QE) was measured by the similar condition, but the light source was changed into high power LED (PLS-LED100B, Beijing Perfectlight Co. Ltd., China). The QE was calculated based on the following equation:
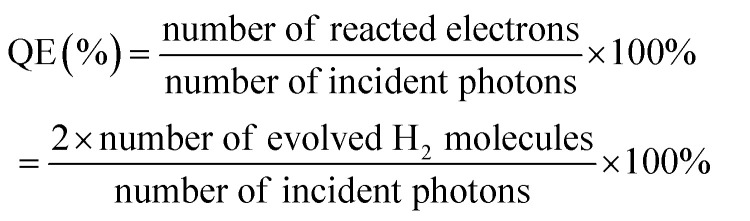


### Photoelectrochemical measurements

The working electrodes were prepared as follow: 0.05 g of the sample, 0.05 g of polyethyleneglycol (molecular weight 20 000) and 0.5 mL of ethanol were mixed and ground into the slurry. Then the slurry was evenly coated onto a 2 cm × 1.2 cm F-doped SnO_2_-coated (FTO) glass electrode by the doctor blade method. The area and the thickness of the coating layer were limited to 1 cm^2^ and 0.01 mm, respectively. Finally, the prepared electrodes were dried in an oven at 100 °C for 60 min. The measurement of the photocurrent and electrochemical impedance spectroscopy (EIS) were performed on an electrochemical analyzer (CHI660E, Shanghai Chenhua Limited, China) in a standard three-electrode system. In this system, the prepared glass electrodes were used as the working electrodes. Pt wire and Ag/AgCl (saturated 0.3 M KCl) were used as counter electrode and reference electrode, respectively. The three electrodes were connected by the electrolyte of 0.5 M Na_2_SO_4_. The source of visible light is a 300 W Xe lamp with a UV cut-off filter (*λ* > 420 nm). The EIS were tested in the same standard three-electrode system, the phase frequency and amplitude-frequency characteristics were recorded over frequency range from 0.01 Hz to 10^5^ Hz, and then the spectrum was transferred into Nyquist diagram.

## Results and discussion

### Structure and properties characterization

**Fig. 1:**
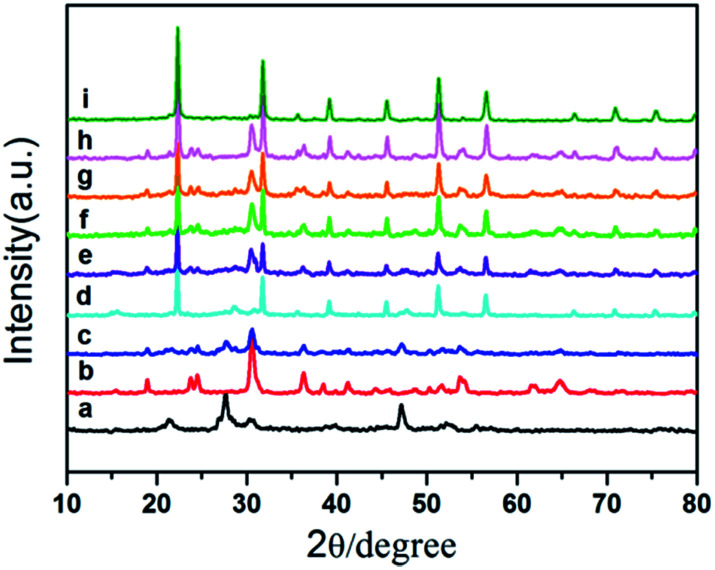
XRD patterns of (a) ZnIn_2_S_4_, (b) ZnWO_4_, (c) ZnIn_2_S_4_/ZnWO_4_, (d) ZnIn_2_S_4_/In(OH)_3_-4.5, (e) ZIZ-3.5, (f) ZIZ-4.5, (g) ZIZ-5.5, (h) In(OH)_3_/ZnWO_4_ and (i) In(OH)_3_.

Fig. 1 shows X-ray diffraction (XRD) patterns of as-prepared samples. The results show that the diffraction peaks of the pure ZnIn_2_S_4_, pure In(OH)_3_ and pure ZnWO_4_ sample are in good agreement with the hexagonal phase of ZnIn_2_S_4_ (JCPDS card No. 03-065-2023), the cubic phase of In(OH)_3_ (JCPDS card No. 76-1464) and monoclinic structure of ZnWO_4_ (JCPDS card No. 73-0554), respectively. For the as-prepared ternary ZIZ composites, all the ZnIn_2_S_4_ and ZnWO_4_ diffraction peaks were found. The characteristic peaks of In(OH)_3_ (22.3°, 31.7° and 51.2°) can also be found. When the pH increases during the synthesis of ZnIn_2_S_4_/In(OH)_3_, the characteristic peak of ZnIn_2_S_4_ at 21.4° becomes weaker, while the peak of In(OH)_3_ at 22.3°, 31.7° and 51.2° become stronger. This demonstrates that the increasing pH value can improve the growth of In(OH)_3_. No traces of other phases were detected, indicating the high purity of the samples and confirming that ZnIn_2_S_4_/In(OH)_3_ and ZnWO_4_ coupled together successfully and without other phases.

**Fig. 2:**
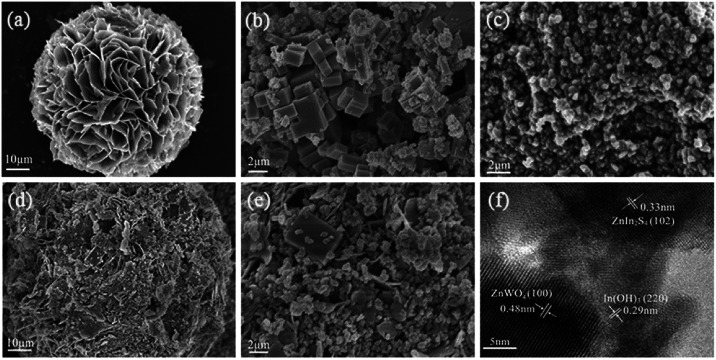
SEM images of (a) ZnIn_2_S_4_, (b) In(OH)_3_, (c) ZnWO_4_, (d and e) ZIZ-4.5, (f) HRTEM images of ZIZ-4.5.

**Fig. 3:**
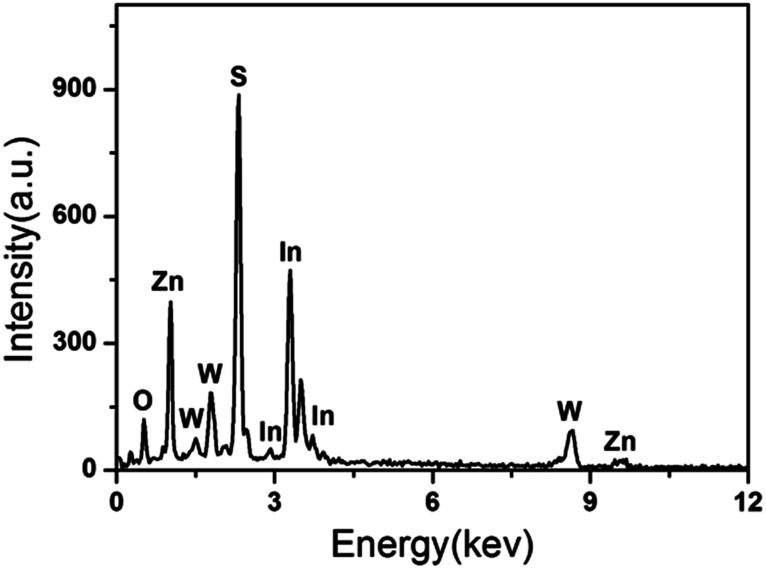
EDS spectrum of ZIZ-4.5 sample.

The morphology of the samples was investigated by SEM. Fig. 2a shows the SEM image of the as-prepared ZnIn_2_S_4_ at a higher magnification. It is shown that the ZnIn_2_S_4_ microsphere has a unique marigold-like spherical superstructure composed of numerous nanosheets. As shown in Fig. 2b, the as-prepared pure In(OH)_3_ was made up of rectangular blocks with an edge of about 0.7–2 μm. It is also clearly seen that some small nanoparticles are attached to the edge of rectangular blocks, indicating that In(OH)_3_ rectangular blocks may be formed by the adsorption of small In(OH)_3_ nanoparticles *via* an Oswald ripening mechanism.^28^ As shown in Fig. 2c, the as-prepared ZnWO_4_ was composed of nanoparticles with a wide size distribution. Fig. 2d and e show the SEM image of the ZIZ-4.5 nanocomposite. It is clearly observed that In(OH)_3_ cubic blocks and ZnWO_4_ nanoparticles were attached to the surface of ZnIn_2_S_4_ microspheres. Fig. 2f shows the HRTEM images of as prepared ZIZ-4.5 sample. As shown in Fig. 2f, the fringes of 0.33 nm, 0.29 nm and 0.48 nm correspond to the (102), (220) and (100) plane of ZnIn_2_S_4_, In(OH)_3_ and ZnWO_4_, respectively.^29–31^ The EDS spectrum (Fig. 3) of the composite clearly confirms the presence of Zn, In, S, O and W elements. From the above results, it can be seen that ZIZ composites with close contact have been successfully fabricated.

**Fig. 4:**
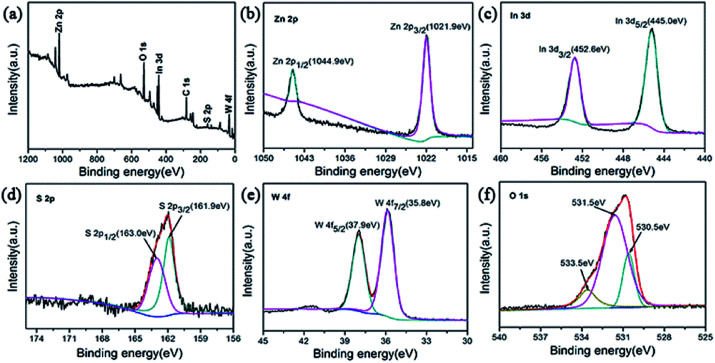
XPS spectra of the ZIZ-4.5 sample: (a) survey, (b) Zn 2p, (c) In 3d, (d) S 2p, (e) W 4f and (f) O 1s.

To characterize the chemical composition of as-prepared ZIZ photocatalyst, X-ray photoelectron spectroscopy (XPS) measurement was used to determine the exact surface state. The XPS survey spectrum confirms that the composite is mainly composed of Zn, In, S, W and O (Fig. 4a). The high-resolution XPS spectra of Zn 2p region of the catalyst was shown in Fig. 4b. The XPS signals of Zn 2p were observed at binding energies at around 1021.9 eV (Zn 2p_3/2_) and 1044.9 eV (Zn 2p_1/2_). The high resolution XPS spectra of In 3d region of the catalyst was shown in Fig. 4c. The XPS signals of In 3d were observed at binding energies at around 445.0 eV (In 3d_5/2_) and 452.6 eV (In 3d_3/2_), which are in good agreement with the previous works.^32^ As illustrated in Fig. 4d, the appearance of S 2p_3/2_ and S 2p_1/2_ peaks at 161.9 and 163.0 eV can be assigned to the S^2−^ in ZnIn_2_S_4_.^33^ The XPS spectrum in the W 4f region exhibits binding energy at 35.8 eV for W 4f_7/2_ and 37.9 eV for W 4f_5/2_ (Fig. 4e), the gap between them is about 2.1 eV, which is close to the value of reference,^34^ indicating that the W element exhibits in the chemical formation of W^6+^. The O 1s region can be fitted by three peaks with binding energies of 530.5 eV, 531.5 eV, and 533.5 eV. The peaks at 530.5 eV and 531.5 eV correspond to the lattice oxygen in ZnWO_4_ and In(OH)_3_,^30,35^ respectively. While the peaks at 533.5 eV can be attributed to the oxygen in the adsorbed H_2_O on the surface of sample. The above results show that the prepared sample is ZIZ.

**Fig. 5:**
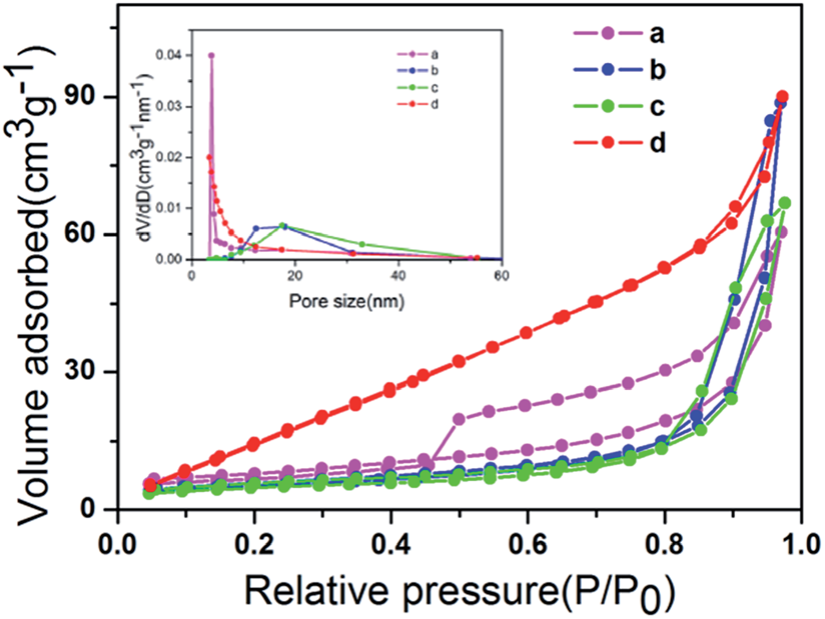
N_2_ adsorption–desorption isotherms of (a) ZIZ-4.5, (b) ZnIn_2_S_4_, (c) In(OH)_3_ and (d) ZnWO_4_. The inset is the corresponding BJH pore size distribution plot.

The nitrogen adsorption–desorption isotherms of ZIZ-4.5, ZnIn_2_S_4_, In(OH)_3_ and ZnWO_4_ are shown in Fig. 5. The obtained results from the sorption isotherms suggest that all samples exhibit a type IV isotherm with a H3 hysteresis loop according to the IUPAC standard,^30,36,37^ indicating the existence of a mesoporous structure or slit-like pore. The porous structure parameters of pure ZnIn_2_S_4_, In(OH)_3_, ZnWO_4_ and ZIZ-4.5 composites are listed in Table 1. The BET surface area (*S*_BET_) of the pure ZnIn_2_S_4_, In(OH)_3_, ZnWO_4_ was 33.730, 13.736, and 11.876 m^2^ g^−1^, respectively. The BET surface area of the ZIZ-4.5 nanocomposite was 27.274 m^2^ g^−1^, which is lower than that of ZnIn_2_S_4_. The reduction of *S*_BET_ in the ternary sample may be ascribed to the blockage of partial macropores due to the surface covering of In(OH)_3_ rectangular blocks and ZnWO_4_ nanoparticles on the bare ZnIn_2_S_4_.

**Table 1:** The BJH pore size, surface areas, pore volumes of the photocatalytic materials

	ZnIn_2_S_4_	In(OH)_3_	ZnWO_4_	ZIZ-4.5
BJH pore size (nm)	3.849	17.500	18.063	3.329
BET surface area (m^2^ g^−1^)	33.730	13.736	11.876	27.274
Pore volume (cm^3^ g^−1^)	0.205	0.142	0.109	0.123

The optical absorption properties of as-prepared samples were evaluated by UV-vis diffuse reflection spectra (DRS) measurement (Fig. S1a†). The pristine ZnWO_4_ showed a narrowed light absorption edge of no more than 380 nm, due to its intrinsic wide band gap.^38^ The pure In(OH)_3_ has weak absorption ability, with an absorption edge of *ca.* 250 nm.^24^ The pure ZnIn_2_S_4_ has a strong absorption in the wavelength ranging from 200 to 550 nm.^39^ The introduction of ZnIn_2_S_4_ increases the light absorption of composite materials in the range of the visible light region. ZIZ-4.5 composite has a strong absorption both in the UV region and visible light region (Fig. S1a†).

The energy level and band gap of the semiconductors play a crucial role in determining their physical properties. The band gap energy of a semiconductor can be calculated by the following formula:

1 *αhν* = *A*(*hν* − *E*_g_)^*n*/2^

where *α*, *h*, *v*, *E*_g_ and *A* are the absorption coefficient, Planck constant, light frequency, band gap energy, and a constant, respectively. Among them, *n* is determined from the type of optical transition of a semiconductor (*n* = 1 for direct transition and *n* = 4 for indirect transition). The *n* value of ZnIn_2_S_4_, In(OH)_3_ and ZnWO_4_ is 1 for the direct transition.^30,40,41^ Therefore, the band gap energy (*E*_g_ value) of the samples can be estimated from a plot of (*αhν*)^2^–(*hν*). The band gap energy of ZnIn_2_S_4_, In(OH)_3_ and ZnWO_4_ is 2.50 eV, 4.99 eV and 3.84 eV, respectively (Fig. S1b†). All the values for the band gap of ZnIn_2_S_4_, In(OH)_3_ and ZnWO_4_ are very close to previously reported results.^42–44^

The valence band edge potential and the conduction band edge potential of a semiconductor material can be determined by using the following equation:^45,46^

2 *E*_VB_ = *X* − *E*^e^ + 0.5*E*_g_

3 *E*_CB_ = *E*_VB_ − *E*_g_

where *E*_VB_ represents valence band edge potential, *X* is the electronegativity of the semiconductor estimated by the geometric mean of the electronegativity of the constituent atoms, *E*^e^ is the energy of free electrons on the hydrogen scale (4.5 eV), *E*_g_ is the band gap energy of the semiconductor, *E*_CB_ is the conduction band edge potential. The *X* value of pure ZnIn_2_S_4_, In(OH)_3_ and ZnWO_4_ is about 4.82, 6.155 and 5.55 eV,^24,44,47^ respectively. The *E*_VB_ of bare ZnIn_2_S_4_, In(OH)_3_ and ZnWO_4_ can be assigned to be +1.57, +4.15 and +2.97 eV, respectively. The corresponding *E*_CB_ of ZnIn_2_S_4_, In(OH)_3_ and ZnWO_4_ can be estimated to be −0.93, −0.84 and −0.87 eV, respectively.

### Photocatalytic activities

**Fig. 6:**
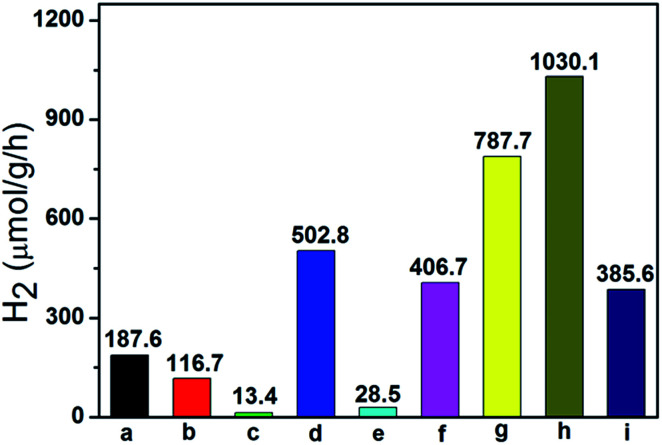
The photocatalytic activity for hydrogen production of (a) ZnIn_2_S_4_, (b) ZnWO_4_, (c) In(OH)_3_, (d) ZnIn_2_S_4_/ZnWO_4_, (e) In(OH)_3_/ZnWO_4_, (f) ZnIn_2_S_4_/In(OH)_3_-4.5, (g) ZIZ-3.5, (h) ZIZ-4.5 and (i) ZIZ-5.5.

Photocatalytic H_2_ production experiments were carried out over these as-prepared photocatalysts in the presence of Na_2_SO_3_ and Na_2_S as the sacrificial reagents under visible light irradiation (*λ* > 420 nm).^48^ As shown in Fig. 6, pure ZnWO_4_ (116.7 μmol g^−1^ h^−1^), pure In(OH)_3_ (13.4 μmol g^−1^ h^−1^) and pure ZnIn_2_S_4_ (187.6 μmol g^−1^ h^−1^) show their low photocatalytic activity, respectively. The ternary ZnIn_2_S_4_/In(OH)_3_/ZnWO_4_ (ZIZ) nanocomposite exhibited enhanced photocatalytic H_2_ evolution activity as compared to all the related pure samples and binary composite photocatalysts. Especially, ZIZ-4.5 shows the highest photocatalytic activity with a H_2_ evolution rate of 1030.1 μmol g^−1^ h^−1^, which was 8.83 times higher than that of ZnWO_4_ alone. Moreover, the apparent quantum efficiency (QE) for H_2_ evolution was examined at 420 nm to further evaluate the photocatalytic activity of ZIZ-4.5. The corresponding QE reached 8.1%, which indicated that the ZIZ-4.5 is a highly-efficient photocatalyst for hydrogen evolution under visible light irradiation. A further increase of pH value will lead to a significant reduction of the photocatalytic H_2_-evolution rate. With increasing pH value, more ZnIn_2_S_4_ transformed into In(OH)_3_ through the hydrolysis reaction of In^3+^ ions, which is evidenced from the XRD measurements (Fig. 1e–g). Excessive amount of In(OH)_3_ particles may cover the active sites on the surface of composite, shield the incident light and act as charge recombination centers.^49^ In addition, the decrease of ZnIn_2_S_4_ can result in the reduction of the utilization of visible light, leading to the decrease of hydrogen production activity.

To confirm the stability of our composite catalysts, we performed four consecutive hydrogen evolution runs over ZIZ-4.5 under the same conditions (Fig. S2†).^50^ Each cycle was performed under visible light irradiation for 3 h. After each run, the reaction system was re-evacuated. The H_2_ evolution rate after the fourth cycle can keep *ca.* 91% of the initial rate. This decrease of the H_2_ evolution rate from 3090 to 2805 μmol g^−1^ after four cycles could be attributed to the continuous consumption of Na_2_S and Na_2_SO_3_.^51^ The above results indicate that the prepared ZIZ-4.5 composite has good stability during photocatalytic reactions.

### Possible mechanism of photocatalytic H_2_ evolution

**Fig. 7:**
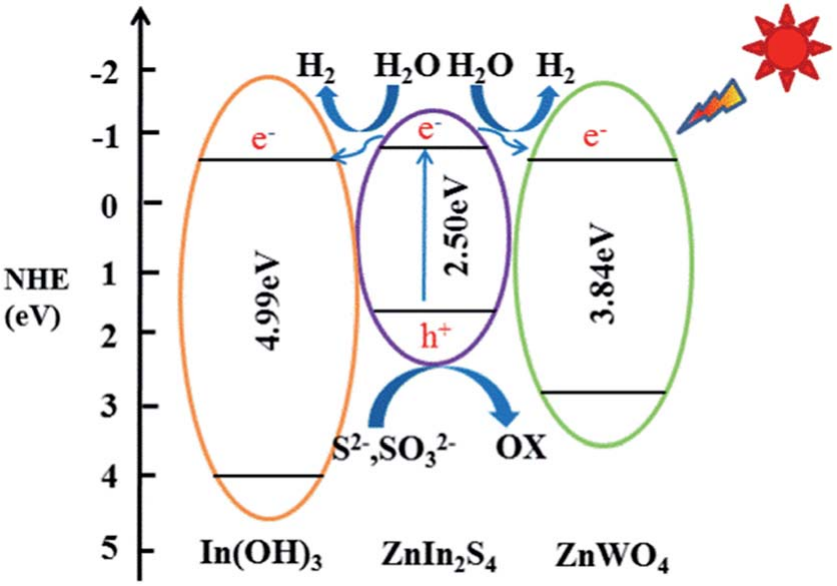
Possible mechanism of the photocatalytic H_2_-production activity of the ternary ZIZ nanocomposite under visible-light irradiation.

A schematic illustration of the tentative mechanism proposed for the high H_2_ production activity of the ternary ZIZ composite is shown in Fig. 7. Under visible light illumination, ZnIn_2_S_4_ with narrow bandgap energy (2.50 eV in this work) can be easily excited and generate electrons and holes. Normally, these photogenerated electrons and holes recombine rapidly, resulting in a poor H_2_ evolution activity of pure ZnIn_2_S_4_. However, in the ternary ZIZ composite system, since the CB of ZnIn_2_S_4_ (−0.93 eV) is more negative than that of In(OH)_3_ (−0.84 eV) and ZnWO_4_ (−0.87 eV), the photogenerated electrons in the CB of ZnIn_2_S_4_ are transferred to the CB of In(OH)_3_ and ZnWO_4_ (Fig. 7), where the electrons can effectively reduce H^+^ and produce H_2_ molecules.^52^ Meanwhile, holes accumulated at the VB of ZnIn_2_S_4_ could be consumed by the sacrificial agent (S^2−^, SO_3_^2−^).^53^ Owning to the close contact of ZIZ composite, photo-generated electron can be transferred quickly, so as to extend the charge carriers lifetime and reduce the recombination of electron–hole pairs. Consequently, the high photocatalytic H_2_-production activity is achieved over the ternary ZIZ composite.

The charge recombination and transfer can be investigated by photoluminescence (PL) spectra. It can also help understanding the fate of photogenerated electrons and holes in semiconductor because PL emission results from the recombination of free carriers.^54^ The PL spectra of ZIZ-4.5, ZnIn_2_S_4_, In(OH)_3_ and ZnWO_4_ samples were recorded at room temperature with an excitation wavelength of 215 nm (Fig. S3†). It can be observed that the bare ZnIn_2_S_4_ shows a strong peak at about 550 nm, which can be attributed to the direct recombination of the photo-generated electron–hole pairs in ZnIn_2_S_4_ nanocrystals. When introducing both ZnWO_4_ and an optimum concentration of In(OH)_3_ content, ZIZ nanocomposite exhibited much lower PL emission peak at about 470 nm. The results implied that ZIZ has remarkably enhanced separation efficiency of photo-induced electron–hole pairs, which is qualitatively in good agreement with the trend in photo-activity enhancement.

To further deeply understand the photocatalytic enhancement mechanism for H_2_ evolution, the transfer and separation of photo-generated charge carriers were also investigated through the photoelectrochemical (PEC) analysis. The transient photocurrent responses of ZnIn_2_S_4_, In(OH)_3_, ZnWO_4_ and ZIZ-4.5 with typical on–off cycles of intermittent irradiation were measured (Fig. S4a†). Notably, the photocurrent of ZIZ-4.5 sample is much higher than the other samples, confirming the more efficient interfacial mobility and separation of photo-generated electron–hole pairs for the ZIZ-4.5 composite sample. In addition, the electrochemical impedance spectroscopy (EIS) has also been investigated to provide sufficient evidence for the interfacial charge transfer resistance and separation efficiency. The electrochemical impedance spectroscopy (EIS) of these four samples were shown (Fig. S4b†). It is observed that the arc radius in the Nyquist plots of ZIZ-4.5 is much smaller than those of ZnIn_2_S_4_, In(OH)_3_ and ZnWO_4_, indicating that a more effective separation of photogenerated electron–hole pairs and a faster interfacial charge transfer had occurred on the surface of ZIZ composite. The PL and PEC analysis results are in good accordance with the photocatalytic-activity measurements, which further confirm that introducing ZnIn_2_S_4_/In(OH)_3_ system is an effective way to improve the photocatalytic efficiency of ZnWO_4_.

**Fig. 8:**
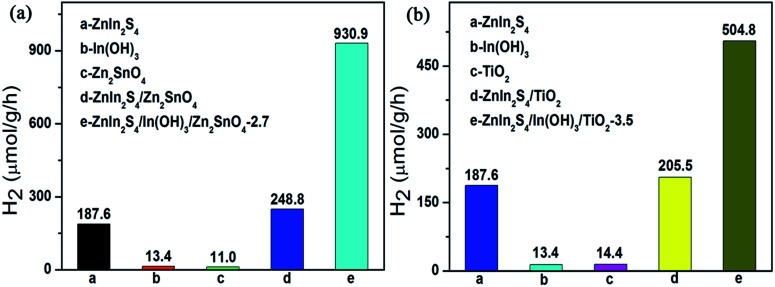
The photocatalytic activity for hydrogen production of (a) ZnIn_2_S_4_, In(OH)_3_, Zn_2_SnO_4_, ZnIn_2_S_4_/Zn_2_SnO_4_ and ZnIn_2_S_4_/In(OH)_3_/Zn_2_SnO_4_; (b) ZnIn_2_S_4_, In(OH)_3_, TiO_2_, ZnIn_2_S_4_/TiO_2_ and ZnIn_2_S_4_/In(OH)_3_/TiO_2_.

Although the results here obtained certainly open up the possibility to design a noble-metal-free photocatalyst for water splitting, the deposition of the ZnIn_2_S_4_/In(OH)_3_ cocatalyst on other semiconductor is certainly required. As shown in Fig. 8, the photocatalytic H_2_-production activity of Zn_2_SnO_4_ and TiO_2_ were both tremendously improved by loading the specified amount of ZnIn_2_S_4_/In(OH)_3_ as cocatalyst. It is expected that the combination of ZnIn_2_S_4_ and In(OH)_3_ as a new class of earth-abundant cocatalyst can become a general strategy to improve the H_2_-evolution activity over various kinds of conventional semiconductors.

## Conclusions

In conclusion, the combination of ZnIn_2_S_4_ with In(OH)_3_ formed a new class of earth-abundant cocatalyst for water-splitting under visible light irradiation, which greatly advanced the photocatalytic activity of ZnWO_4_ promoted by synergetic effects. The roles of ZnIn_2_S_4_ and In(OH)_3_ in boosting the H_2_ evolution activity of ZnWO_4_ were revealed. This work could not only provide an excellent candidate for visible-light H_2_ evolution, but also opens new possibilities to provide insight into the design of new system with high activity for solar hydrogen generation.

## Conflicts of interest

There are no conflicts to declare.

## Supplementary Material

RA-008-C7RA12586K-s001
