# Supplementary material for: Cocatalyst designing: a binary noble-metal-free cocatalyst system consisting of ZnIn2S4 and In(OH)3 for efficient visible-light photocatalytic water splitting
Source: RSC Adv. 2018 Jan 29;8(9):4979–86. doi: 10.1039/c7ra12586k (PMC9078037; doi:10.1039/c7ra12586k)
Supplement: RA-008-C7RA12586K-s001 [file RA-008-C7RA12586K-s001.pdf]

Electronic Supplementary Material (ESI) for RSC Advances.
This journal is © The Royal Society of Chemistry 2018

Cocatalyst Designing: A Binary Noble-metal-free Cocatalyst System Consisting of $ZnIn_2S_4$ and $In(OH)_3$ for Efficient Visible-light Photocatalytic Water Splitting

Jinyan Zhao [a], Xiaoming Yan [a], Ning Zhao [b], Xiao Li [a], Bin Lu [a], Xuhong Zhang*[,a] and Haitao Yu*[,a]

College of Chemistry and Materials Science, Hebei Normal University , Shijiazhuang

050024,China

**To whom correspondence should be addressed.*
The address for corresponding author
Xuhong Zhang:
College of Chemistry and Materials Science, Hebei Normal University, Shijiazhuang 050024,China
Email address: xuhongshd@126.com
Telephone: +86-311-8078-7400
Haitao Yu:
College of Chemistry and Materials Science, Hebei Normal University, Shijiazhuang 050024,China
Email address: haitaoyu@hebtu.edu.cn
Telephone: +86-311-8078-7400

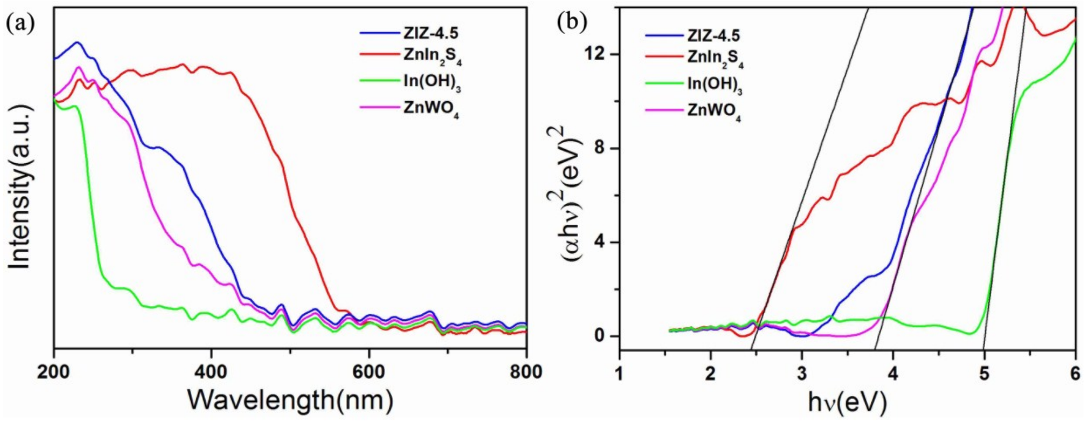

Fig. 1 (a) UV-vis diffuse reflectance spectra of ZIZ-4.5, $ZnIn_2S_4$, $In(OH)_3$ and $ZnWO_4$, (b) The band gap energy of $ZnIn_2S_4$, $In(OH)_3$ and $ZnWO_4$.

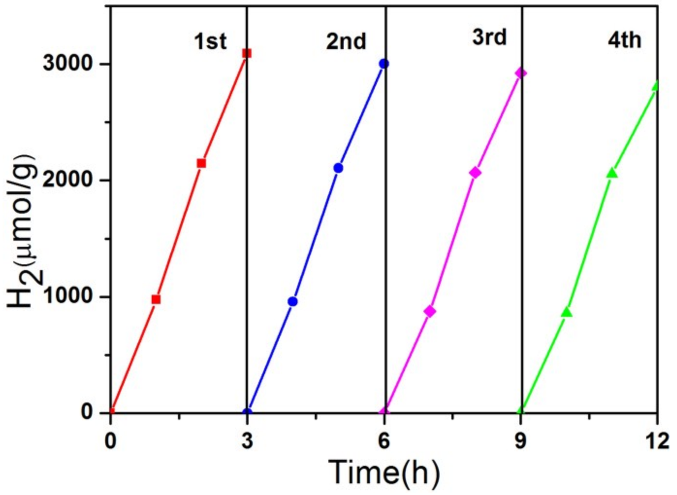

Fig. 2 Cyclic H_2 production on ZIZ-4.5 photocalyst. Reaction Conditions: photocatalyst 50 mg; light source, 300 W Xenon lamp equipped with a cut-off filter (λ > 420nm); Reactant solution: 100 mL aqueous solution containing 0.25M Na_2SO_3 and 0.35M Na_2S.

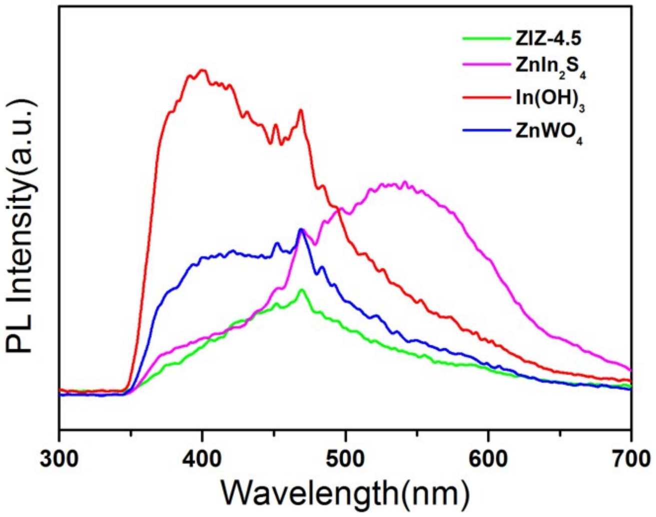

Fig. 3 Photoluminescence (PL) spectra of ZIZ-4.5, $ZnIn_2S_4$, $In(OH)_3$ and $ZnWO_4$ at room temperature (λex = 215 nm).

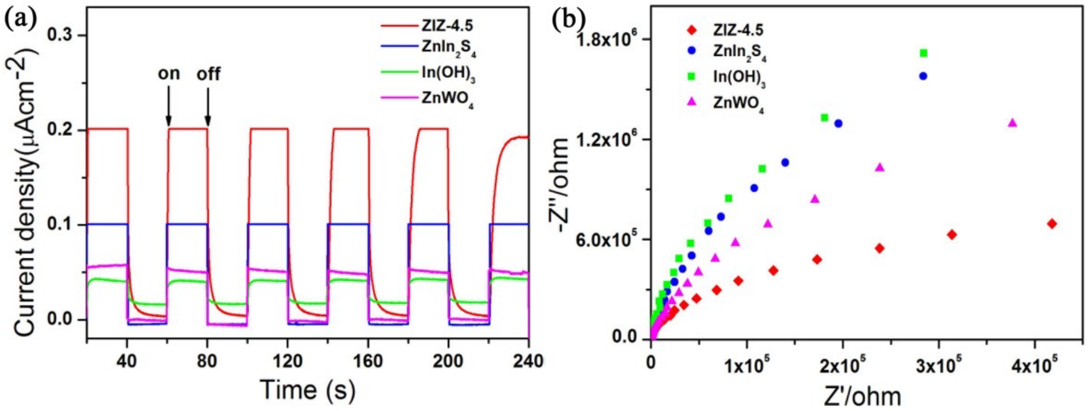

Fig. 4 (a) Transient photocurrent response and (b) the electrochemical impedance spectroscopy of ZIZ-4.5, $ZnIn_2S_4$, $In(OH)_3$ and $ZnWO_4$.
